# Tailoring the thermal conductivity of the powder bed in Electron Beam Melting (EBM) Additive Manufacturing

**DOI:** 10.1038/s41598-017-11243-8

**Published:** 2017-09-05

**Authors:** C. J. Smith, S. Tammas-Williams, E. Hernandez-Nava, I. Todd

**Affiliations:** Department of Material Science & Engineering, The University of Sheffield, Sir Robert Hadfield Building, Mappin St, Sheffield, S1 3JD UK

## Abstract

Metallic powder bed additive manufacturing is capable of producing complex, functional parts by repeatedly depositing thin layers of powder particles atop of each other whilst selectively melting the corresponding part cross-section into each layer. A weakness with this approach arises when melting overhanging features, which have no prior melted material directly beneath them. This is due to the lower thermal conductivity of the powder relative to solid material, which as a result leads to an accumulation of heat and thus distortion. The Electron Beam Melting (EBM) process alleviates this to some extent as the powder must first be sintered (by the beam itself) before it is melted, which results in the added benefit of increasing the thermal conductivity. This study thus sought to investigate to what extent the thermal conductivity of local regions in a titanium Ti-6Al-4V powder bed could be varied by imparting more energy from the beam. Thermal diffusivity and density measurements were taken of the resulting sintered samples, which ranged from being loosely to very well consolidated. It was found that the calculated thermal conductivity at two temperatures, 40 and 730 °C, was more than doubled over the range of input energies explored.

## Introduction

Powder bed fusion (PBF) additive manufacturing (AM) has proven capable of fabricating complex and functional geometries with mechanical properties similar to those of wrought origin (the reader is referred to a review paper for more detail^1^). AM will build a part from the ground up by first decomposing it into a stack of thin cross-section slices (typically between 20 and 100 µm); during PBF these cross-sections are then melted into layers of powder, sequentially deposited one atop of another. The two predominant heat sources used to melt the powder are laser (commonly referred to as selective laser melting or SLM) and electron beam (referred to as electron beam melting or EBM). Although the methodology of both approaches is near identical, the electron beam process presents additional complications in that a negative charge will be induced in powder particles exposed to the beam. The subsequent repulsive force can become so great as to cause a powder cloud as particles are repelled from the bed. This necessitates the need for the powder bed to be lightly sintered before melting. Fortunately, this can be performed by the electron beam itself owing to its very high deflection speeds. By defocusing the beam and scanning it across the bed at high speed repeatedly, enough energy can be imparted to sinter the powder without inducing an excessive electrical charge. The degree of sintering required will depend on the electrical conductivity of the material being melted. Titanium Ti-6Al-4V for instance, the material of focus in this paper, has a relatively low electrical conductivity when compared with other metals. In the standard Arcam process for Ti-6Al-4V sintering is conducted in two stages. First, the whole bed is lightly sintered (dubbed Preheat I by Arcam), before the area to be melted is sintered to a greater degree (Preheat II).

Although the need to sinter the powder bed prior to melting can be perceived as a disadvantage compared with the SLM process it does introduce two major benefits. The first is that by selectively melting into a powder bed at an elevated temperature, the temperature difference between the melt area and the surrounding powder is lower reducing the amount of residual stresses that occur due the fast cooling rate^2^. Moreover, because the bed remains hot during the build any residual stresses that may have been introduced during melting are effectively relieved. As a result parts made using EBM should not require stress relieving heat treatments post-build^3, 4^. Numerous researchers have tried to replicate these desirable traits with SLM by using resistance heaters^5^. Secondly, the increased connectivity of the sintered powder relative to loose powder will improve the thermal conductivity of the powder bed. This becomes a desirable trait when melting over-hanging features (or negative surfaces) which, as the name suggests, have no prior material melted beneath them. These features are generally a weakness of all powder bed additive manufacturing processes as the thermal energy imparted during melting is not as quickly dissipated by the powder as it would be by solid, prior melted material^6^. This problem is exacerbated when the powder bed has not been sintered. Further, an increased powder bed density has been shown, at least numerically, to result in a more defined melt pool^7^.

Several studies have characterised the thermal conductivity of sintered powder and solid parts made from Ti-6Al-4V using EBM; a summary of these is shown in Table 1. The conductivity of Solid Ti-6Al-4V has been reported to be between 6.2 and 7.1 W/mK at room temperature and between 10 and 15 W/mK at 700–750 °C, which is around the operating temperature of the process. Loose and sintered Ti-6Al-4V powder (45–106 µm particle size) has been reported to be approximately 10% of that at room temperature. However, there appears to be some inconsistencies with these studies as one reports a higher relative density and thermal conductivity of the loose powder than that of sintered powder. It can be seen in the last three entries of Table 1 that reported values for the bulk density of Ti-6Al-4V powder do vary considerably, highlighting the difficulty in measuring the bulk density of sintered powder accurately.

**Table 1:** Values of thermal conductivity and density of Ti-6Al-4V from the literature.

Relative density (%)	Origin	Reported thermal conductivity (W/m·K)		
		25 °C	700 °C	750 °C
100 (solid)	Wrought^8^	6.2	—	10
	Wrought^9^	7.0	15	—
	Wrought^10^	—	12.5	13.1
	EBM-PREP^11^	6.7	13.7	14.5
	EBM-GA^11^	7.1	14.6	15.3
50.0	EBM-GA (sintered)^8^	0.63	—	2.44
58.7	EBM-GA (loose)^11^	0.79	0.96	1.25
56.4	EBM-PA (sintered)^12^	—	—	—

Relative density is based on a value of 4.43 g/cc for Ti-6Al-4V. EBM entries used powder produced by Gas Atomisation (GA), Plasma Atomisation (PA) or the Plasma Rotating Electrode Process (PREP), all yielding a particle size range of 45–106 µm.

This paper presents a more comprehensive study of thermal conductivity measurements of Ti-6Al-4V powder (45–106 µm) sintered using a range of beam input energies. Samples were extracted from and where thermal diffusivity and density measurements were taken. The resulting measurements are related to the beam parameters and thus allow the energy/beam settings used to sinter the powder to be directly correlated to the thermal properties of the powder, which is not currently possible. Not only will this enable more accurate inputs to models of melt pool behaviour but could allow the level of thermal conductivity to be tailored for local requirements.

## Methods

### Selectively sintering samples using EBM

**Figure 1:**
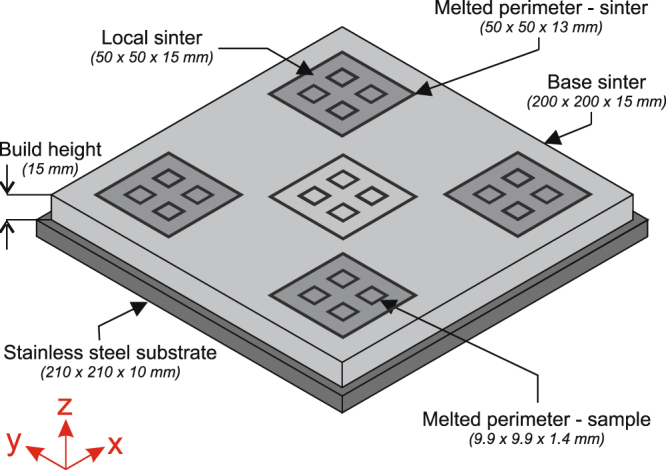
Layout of samples during EBM build.

Sintered powder samples produced using nine different beam input energies were evaluated; these were produced over two builds. In each build, the whole 200 × 200 mm area was first lightly sintered (referred to as base sinter and analogous to preheat I in the Arcam process) and then four 50 × 50 mm areas were further sintered to varying degrees (referred to here as local sintering and analogous to preheat II in the Arcam process). To permit samples of approximately the same dimensions to be extracted from each of the sintered regions, a square perimeter was melted into the sintered powder with a single melt track (~0.7 mm wide). As well as controlling the dimensions of the sample, it also provided structural integrity during handling and testing, particularly for the more lightly sintered samples. Four sample perimeters were melted into the base sinter and each of the local sinter regions, which is illustrated in Fig. 1. Melted perimeters were also created around each 50 × 50 mm sintered region (including the base sinter) to permit a block of sintered powder of known volume to be extracted intact and weighed for density calculation. These melted perimeters started at 2 mm above the stainless steel substrate to allow easy removal.

Typically, the Arcam process uses a one-dimensional thermal model to approximate how much energy input is required during preheating and melting phases to maintain the temperature of the current layer and ensure the powder bed is adequately sintered. This model was disabled, as it would have varied the energy input from the beam as the build progressed. Instead, a fixed energy was applied for the base sinter and each local sinter region throughout the build. This then allows the beam energies to be directly related to the measured thermal diffusivity and density. The beam parameters for both the base and local sinter regions are shown in Table 2. The number of times the base and local areas were scanned with these beam parameters are shown in Table 3 i.e. the beam rastering across the whole area is defined as one pass. The total energy imparted based on the number of passes is also calculated. Note that for both builds the total energy delivered by the electron beam into the deposited powder, neglecting any effect of coupling, was identical for both builds.

**Table 2:** Beam parameters for base and local sintering.

Beam parameters	Base sinter	Local sinter
Current (mA)	30	38
Velocity (mm/s)	14600	14600
Line Spacing (mm)	1.2	1.2
Line Order	15	15
Focus offset (mA)	50	50
No. of passes	4	***Varied***
Dimensions (mm)	200 × 200	50 × 50

**Table 3:** Repetitions and calculated energies for both the base and local sinter regions.

Sinter location	Build 1			Build 2		
	No. of passes	Line Energy (J/m)	Total Energy (kJ)	No. of passes	Line energy (J/m)	Total Energy (kJ)
***Base***	***4***	***123.29***	**16438.4**	***4***	***123.29***	**16438.4**
1	1	156.16	325.3	2	156.16	650.7
2	7		2277.4	8		2602.7
3	6		1952.1	5		1626.7
4	4		1301.4	3		976.0
5	0		0.0	0		0.0
Total			22294.5			22294.5

Total energy is for the sinter area in each layer. Note that energies stated are from the beam and do not account for coupling effects with powder.

The powder feedstock was supplied by Arcam and consisted of pre-alloyed, plasma atomised Ti-6Al-4V. The nominal size range was given as 45–106 µm. A single batch of powder was used for all experiments to avoid any variation due to chemistry/morphology. The layer thickness, i.e. the movement of the build plate downwards before new powder was deposited, was set at 70 µm.

### Measuring thermal diffusivity using the laser flash method

The thermal diffusivity for each sample was measured in the vertical (z), build direction and recorded at two temperatures, 40 and 730 °C. The former allowed the furnace control software to maintain a highly consistent temperature while still giving properties representative of those that would be acquired at room temperature. The latter is the temperature the Arcam EBM process attempts to maintain the powder bed at, when standard settings are applied for manufacture of Ti-6Al-4V components. The temperature was increased using an infrared heater by 5 °C/min until the target temperature was reached. The allowable temperature variation was <1 °C for 11 minutes prior to taking a measurement.

The thermal diffusivity of each sinter sample was measured using an Anter Flashline 3000 which applies the laser flash method. A high speed xenon discharge tube provided a transient energy pulse and a liquid nitrogen cooled infrared detector measured the temperature response of the sample. The thermal diffusivity is determined by measuring how long it takes the energy imparted from the xenon lamp to pass through the sample and reach the other side. The simplest model for determining the thermal diffusivity is that by Parker^13^, which assumes one dimensional adiabatic heat flow. The thermal diffusivity (α) is calculated from the sample thickness (*z*_*t*_) and the time taken to reach half the maximum temperature increase (t_0.5_) using Eqn (1). This model is valid provided heat losses and finite pulse times are a minimum.

1 $$\alpha =0.1388\cdot \frac{{{z}_{t}}^{2}}{{t}_{0.5}}$$

**Figure 2:**
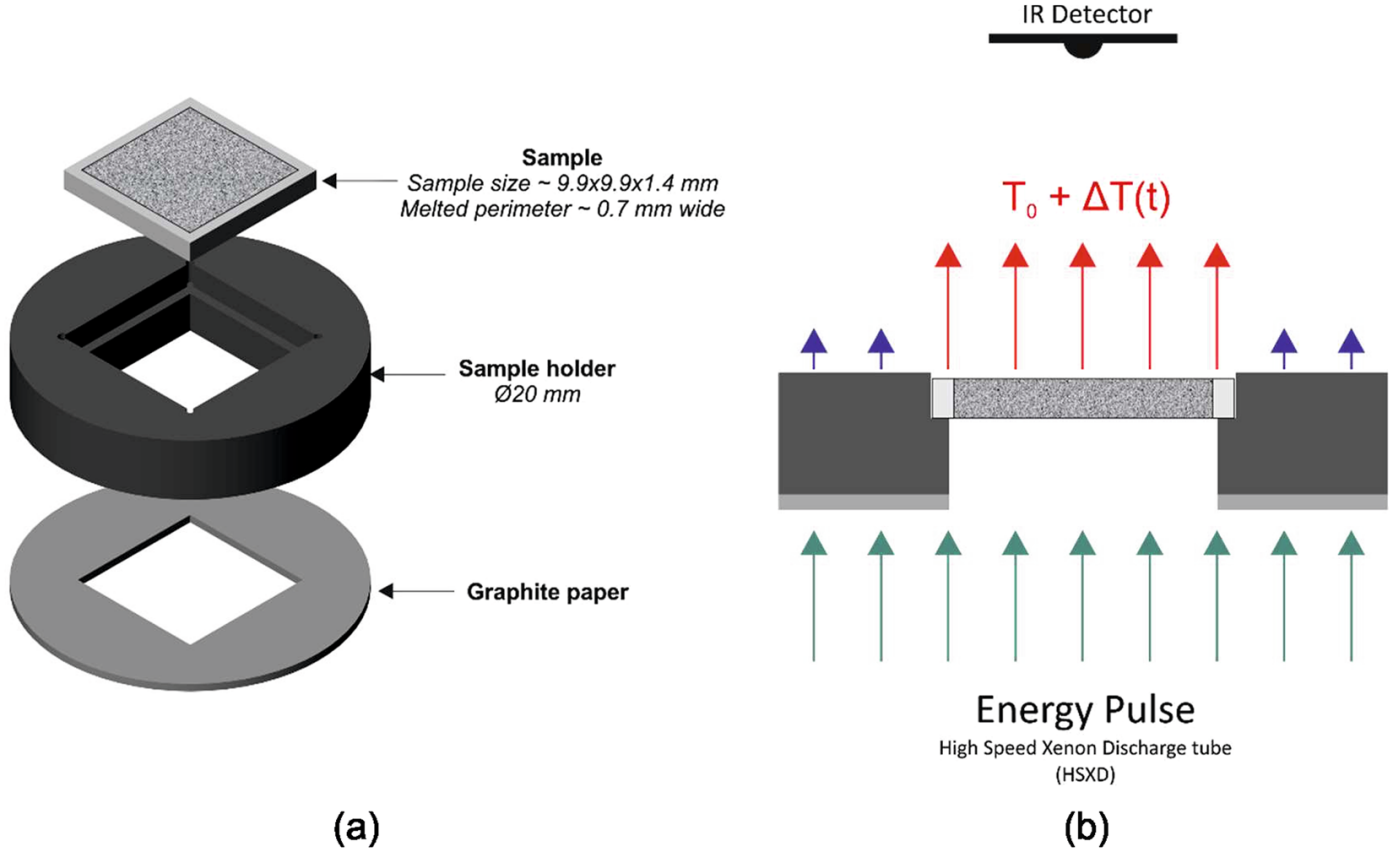
(**a**) Assembly of sample, sample holder and graphite paper for insulation (**b**) A 2-D schematic of the sample in the sample holder being exposed to an energy pulse with the resulting increase in temperature (ΔT) measured by an Infrared sensor. T_0_ is the furnace temperature, which was set to 40 and 730 °C for each test.

The testing configuration of each sample and a schematic of how the laser flash method is applied to the sample is shown in Fig. 2. The laser flash equipment is capable of testing two samples simultaneously, which were selected to be from the same sinter region to quantify variation introduced by sample preparation. Each sample was measured three times in succession to attain an average and to quantify the measurement errors. The same sample thickness was used for all tests, selected to be approximately 1.4 mm as this should result in a half max time between 10 and 1000 ms, as recommended by ASTM E1461–13.

### Measuring sample porosity

The sinter blocks were carefully extracted and the bottom face of the sinter block surface was made flush with the melted perimeter by using grinding paper (P180) on a flat surface. The mass of each sinter block was then recorded. The powder was then removed and the inner length and width of the melted perimeter as well as the height was measured at multiple points with callipers and averaged. The mass of the melted perimeters were then measured and subtracted from the total mass. The volume of the sample perimeters were calculated using a density balance and subtracted from the total volume.

To complement the density values calculated for each of the sintered blocks, two of the small samples from each sintered block were also characterised using a gravimetric approach i.e Archimedes method. This is challenging with open porosity as the measuring fluid will infiltrate the samples. Coating the samples in an impermeable, robust medium of known density (such as paraffin wax) is a potential solution although it is difficult to ensure it adheres to the surface of the sample without infiltrating it (if applied in a liquid state) or trapping pockets of air. Mercury porosimetry is a potentially attractive approach as mercury will not infiltrate pores smaller than ~360 µm under vacuum conditions. As the pressure is increased infiltration will occur first with larger pores and then eventually filling the smallest (~3 nm)^14^. Unfortunately, the sintered samples analysed in this study have very little structural integrity and the high pressures (eventually reaching ~345 KPa) would have likely damaged the samples. An alternative approach is to fully saturate the porous regions samples with the same fluid medium used for the hydrostatic balance and measure the total mass. This method is commonly used for porous media such as rocks and aggregate, with the latter covered under ASTM C127 and C128 standards.

No standard exists for sintered metallic powder, so ASTM C128 (relative density for fine aggregates) was broadly followed. Samples were first kept under vacuum (10^–4^ mbar) for an 18 hour period to remove moisture. The mass of each sample was then measured which corresponded to the dry mass (M_d_). Each sample was then saturated in distilled water over a 72 hour period to allow the capillary effect to displace the air in the pores. The mass of the sample was then measured again which corresponded to the saturated mass (M_sat_). The mass of the saturated samples were then measured on a hydrostatic balance immersed in distilled water, yielding the immersed mass (M_I_). The relative density (*ρ*_*r*_) of the sample was then calculated using Eqn (2).

2 $${\rho }_{r}=\frac{{M}_{d}}{{M}_{sat}-{M}_{I}}$$

Qualitative assessment of the morphology of the porosity was conducted using an FEI InspectF SEM. Samples were sectioned to allow examination of both the x-y and x-z planes.

## Results

### Thermal diffusivity

**Figure 3:**
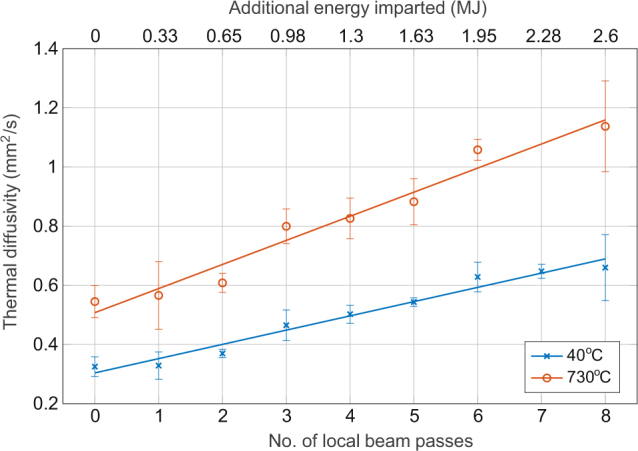
Thermal diffusivity of the sintered samples with increasing number of local beam passes. The energy imparted based on the number of beam passes is also shown. Error bars correspond to the standard deviation.

The measured thermal diffusivity displays a clear positive correlation with the number of additional passes of the beam, which is also shown as an energy in Fig. 3. For both temperatures tested a linear trend line has been fitted by linear regression and appears to adequately describe the relationship, with an R^2^ of 0.97 and 0.96 for the high and low temperature respectively.

### Density

**Figure 4:**
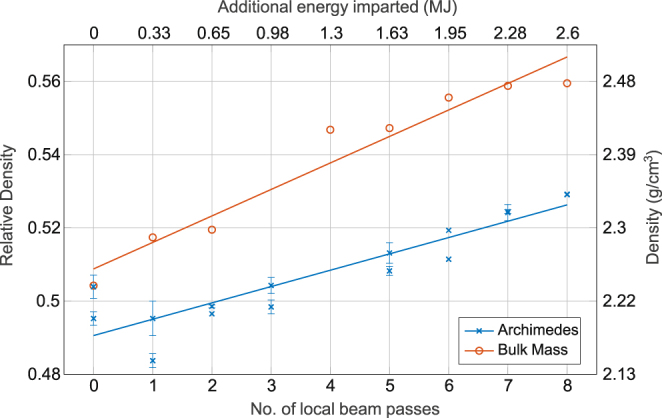
Measured density of the sintered samples with increasing number of local beam passes. The energy imparted based on the number of beam passes is also shown. Error bars correspond to the standard deviation.

The relative and measured density also exhibits a positive linear relationship with the number of additional passes of the beam when measured by both methods, as seen in Fig. 4. Trend lines fitted by linear regression yielded R^2^ values of 0.97 and 0.84 for the direct mass measurement and saturated Archimedes method datasets respectively. The relative densities as determined by the saturated Archimedes approach were between 2 and 4% lower than those determined by direct mass measurements. The overall rise however, as determined by both methods, was fairly small changing <6% over the range of local beam passes.

## Thermal conductivity

**Figure 5:**
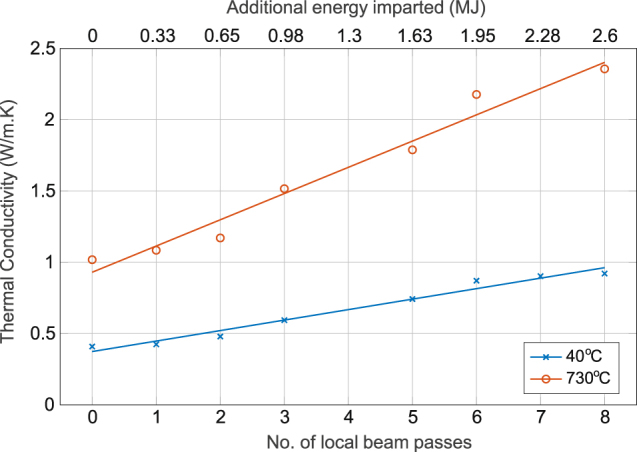
Calculated thermal conductivity of sintered samples with increasing number of local beam passes. The energy imparted based on the number of beam passes is also shown. The thermal conductivity was calculated from the measured thermal diffusivity and density values.

The thermal conductivity (k) was calculated from the mean thermal diffusivity (α) and mean density (ρ) for each local beam pass using Eqn (3). Literature values of 0.563 and 0.836 J/gk were used as the heat capacity (C_p_) for 40 and 730 °C respectively^15^. The resulting plot is shown in Fig. 5.

3 $$k=\rho \alpha {C}_{p}$$

The densities as determined from direct mass measurements of each sinter block were used in calculating the thermal conductivity. The thermal conductivity values of porous Ti-6Al-4V from the literature (Table 1) fall within the range calculated here for both temperatures.

### Electron microscopy

**Figure 6:**
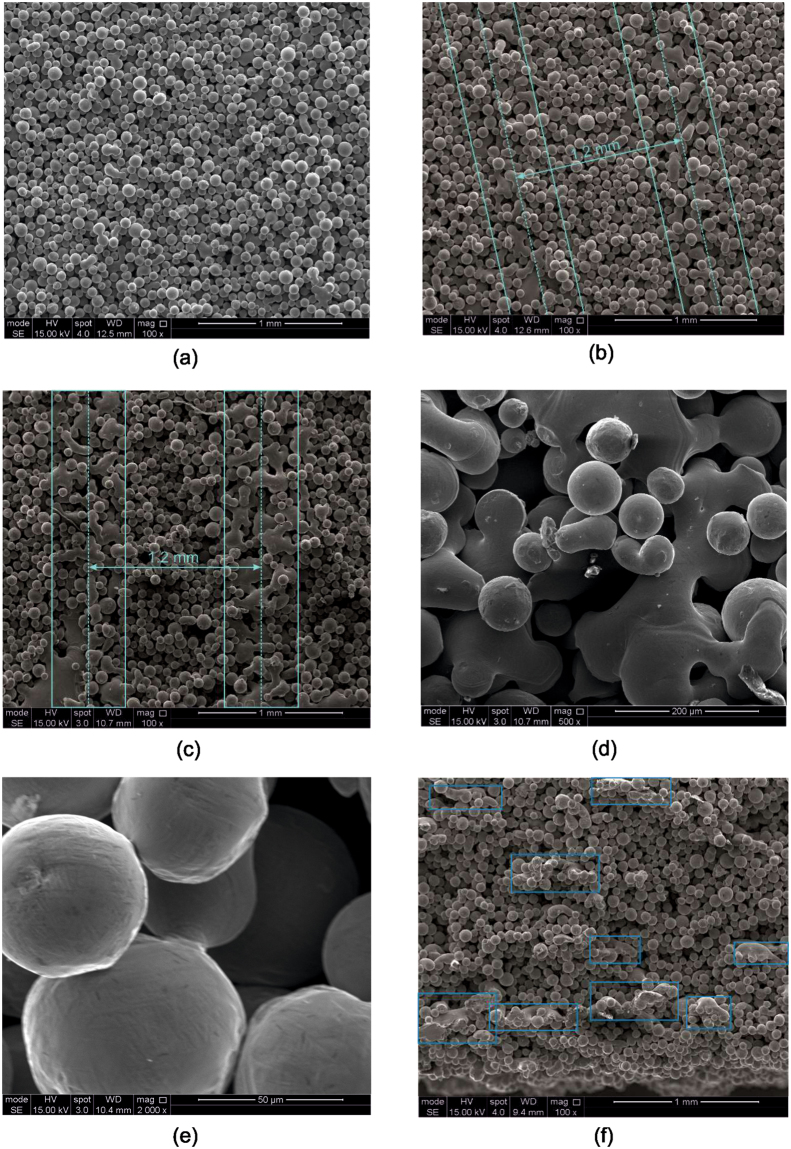
SEM images of sintered samples. (**a**) Sample with one local beam pass, (**b**) Sample with four local beam passes, (**c**) Sample with eight local beam passes, (**d**) High magnification image of melted region – eight local beam passes, (**e**) High magnification image of sintered powder particles – eight local beam passes and (**f**) Section in the build direction – eight local beam passes with partially melted regions highlighted. Images (**a**) to (**e**) show the x-y plane while (**f**) shows the x-z plane (across the build layers).

Macroscale SEM analysis of the sintered powder revealed that the sintering was not uniform for any of the samples, with the regional variation in each sinter appearance becoming more noticeable as the number of beam passes was increased (Fig. 6a,c). Indeed, at higher numbers of beam passes lines of partially melted particles are clearly visible (Fig. 6b,c). It is apparent that the distance between such regions corresponds to the line spacing defined during setup. Higher resolution images of the melted regions (Fig. 6d) confirms that the powder has melted, rather than formed the necks typically associated with sintering processes. In contrast, no melting can be observed in other regions (Fig. 6e). Images of the sinter taken to view the x-z plane (Fig. 6f) show partially melted regions, which are all elongated perpendicular to the build (z) direction.

## Discussion

There is a strong linear correlation between the number of local beam passes and the thermal diffusivity. It can be seen that over this range of input energies the thermal diffusivity more than doubles when measured at both 40 and 730 °C. In contrast, the relative density only changes by around 4%, which implies that the thermal conductivity is mostly influenced by changes to the morphology rather than the densification of the sinter. In particular, the connectivity between the powder particles increases with more local beam passes (Fig. 6a,c) by two mechanisms, sintering (Fig. 6e) and localised melting (Fig. 6d). This is consistent with what has been reported in the literature previously^16, 8^, which corresponds to where the beam has passed. At the highest energy there is a great deal of partial melting occurring, specifically where the beam has passed as observed from the tracks which, appear to be spaced ~1.2 mm apart (corresponding to the line spacing of the beam). Figure 6e demonstrates that the depth of these tracks is very shallow. This could imply that the thermal conductivity would be greater if measured in the x or y direction, instead of the z measured here. Moreover, when modelling the behaviour of the melt pool sit may be prudent to include the inhomogeneous sinter that has been observed here, or at least recognise that the sinter may have periodic variation.

The excessive partial melting seen in the higher energy samples is not ideal as it will reduce the recyclability of the powder and make it harder to remove post-build. This proved to be the case here, with the samples produced using seven and eight local beam passes the powder was almost impossible to remove by bead blasting. Reducing the line energy and the beam speed, which would impart less energy and allow more time for heat to dissipate before the next pass could help alleviate this problem. This would of course require more beam passes to impart the same amount of energy albeit over a longer time period. Perhaps a more ideal solution would be to optimise the path of the beam so that the energy is more evenly distributed across the layer. The current back and forth scanning strategy used by the Arcam EBM process involves the beam always travels along the same concentrated tracks with each pass, meaning that much of the powder bed is never directly exposed to the beam. An optimised beam path would not only ensure all of the bed is exposed to the beam, but also that the time before the beam returns to the same or nearby location is maximised. This may result in achieving an increase in particle connectivity without excessive melting.

The relative density changes by both direct mass measurement and the water saturation method showed a change of <6% over the range of input energies. Although showing a similar trend to the direct mass measurements, the relative density determined using the water saturation method was between 2 and 4% lower. This may indicate that not all the air was displaced by water during the saturation phase. It does however highlight the potential of the water saturation method for at least estimating the density of sintered metal powder, particularly if the volume cannot easily be calculated from the geometry.

It is envisioned that this approach of increasing the thermal conductivity of the powder bed would only the applied in the vicinity of powder to melted rather than the whole bed. The rest of the powder bed would remain loosely consolidated to allow for easy recovery and recycling of the powder post-build.

## Conclusions

The measured thermal diffusivity more than doubled over the range of local beam passes explored whereas the relative density changed by just 4%. Thus, the resulting change in thermal conductivity is almost exclusively related to a morphological change rather than densification of the powder itself. Electron microscopy revealed that with more local beam passes there was an increase in the connectivity of the powder particles through both necking and partial melting. Partial melting was mostly observed in regions where the beam had scanned over, and was more evident in samples with an increasing number of passes. Partial melting is undesirable for powder recycling; future work should focus on trying to maximise particle connectivity with minimal melting, if an increase in thermal conductivity is desired. This would involve optimising the beam scanning strategy, which at the very least would avoid the beam travelling along the same path on subsequent passes.
